# Anatomical Study of Chiari Network and the Remnant of Left Venous Valve in the Interior of Right Atrium

**DOI:** 10.1155/2015/247680

**Published:** 2015-09-09

**Authors:** D. Devi Jansirani, S. Shiva Deep, S. Anandaraja

**Affiliations:** ^1^Department of Human Structure and Neurobiology, Oman Medical College Affiliated to West Virginia University, 321 Sohar, Oman; ^2^Department of Radiodiagnosis, Badr-Al-Samaa Polyclinic, 112 Sohar, Oman; ^3^Department of Cardiology and Cardiac Electrophysiology, Indira Gandhi Government General Hospital and Postgraduate Institute, Puducherry 605 001, India

## Abstract

Chiari network occurs due to incomplete resorption of right valve of sinus venosus. It is often noticed as fenestrated membranous structure or reticular network like structure in the valve of inferior vena cava and coronary sinus. The remnant of left venous valve is observed as trabeculae over the fossa ovalis. The incidence of Chiari network and the remnant of left venous valve were studied in 80 cadaveric hearts utilized for teaching the undergraduates. The right atrium was opened anterior to sulcus terminalis and the interior was examined for the presence of these embryological remnants. The incidence of Chiari network and left venous valve in the present study is 3.75% and 7.5%, respectively. Chiari network was observed as a fenestrated membranous structure in 2 specimens and a reticular network in 1 specimen, with variable extension to coronary sinus opening and right atrial wall. The remnant of left venous valve was observed as multiple fine strands in 3 specimens and trabecular structure in 3 specimens. These structures may create diagnostic confusion, difficulty in interventional procedures, and complications like thromboembolic events. Hence, the knowledge about the incidence, morphology, and clinical manifestations of these rare embryological remnants is mandatory.

## 1. Introduction

In 1897, Hans Chiari described abnormal fibrous lace-like strands extending from the margin of the inferior vena cava or coronary sinus valves to the crista terminalis region. The network was termed after him. The Chiari network is derived from the incomplete resorption of right valve of sinus venosus [1]. The other valve, left venous valve, usually fuses with atrial septum. However, it may be incompletely resorbed leaving a trabecular remnant over the fossa ovalis [2].

The study or case report mentioned in most of the literature about these embryological remnants was based on echocardiographic findings. There is paucity of literature about the anatomical study of these structures. Although Chiari network was considered as a benign variant in the far past, the use of echocardiography allowed recognition of the network with its associated complications [3]. It also possesses diagnostic confusion since it mimics other pathological manifestations [4]. The remnant of left venous valve is even less often discussed. However, its interference complicating the interventional procedure had been reported [5].

Hence, the aim of this study is to find the incidence of these rare embryological remnants with its morphology and possible associated clinical manifestations.

## 2. Material and Methods

A total of 80 heart specimens collected for the purpose of teaching undergraduate students were utilized for this study. These specimens were collected irrespective of age, sex, and race from Department of Anatomy, Sree Gokulam Medical College, India, and Department of Anatomy, Oman Medical College (affiliated to West Virginia University), Oman. The specimens from Oman Medical College were provided by Department of Anatomy, West Virginia University, United States.

The heart was opened anterior to sulcus terminalis. The interior of right atrium was observed for the presence of Chiari network and remnant of left venous valve.

## 3. Observation

Out of 80 hearts studied, Chiari network was noticed in 3 specimens (3.75%) and remnants of left venous valve were noticed in 6 specimens (7.5%).

### 3.1. Chiari Network

**Figure 1:**
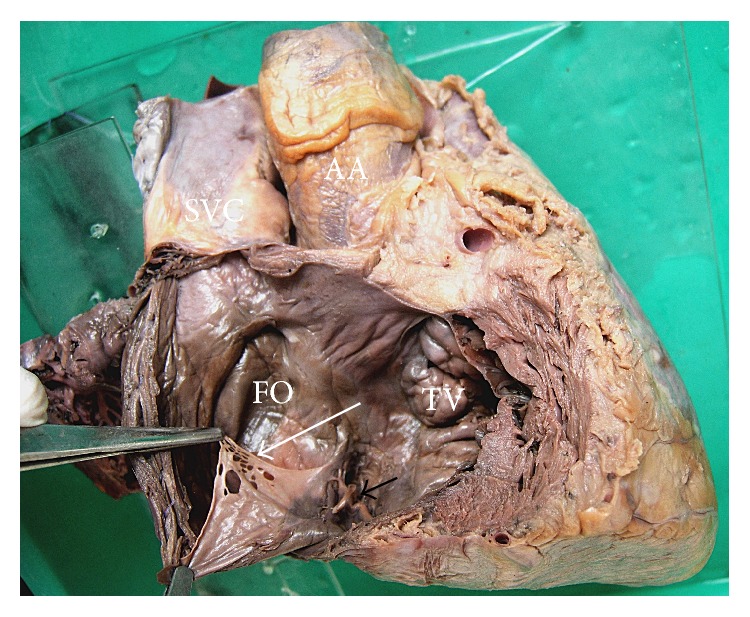
Interior of right atrium showing Chiari network as a fenestrated membranous structure (*long white arrow*) involving the valve of inferior vena cava, with fibrous strands extending to the coronary sinus opening (*short black arrow*).* SVC: superior vena cava*,* AA: ascending aorta*,* FO: fossa ovalis*,* and TV: tricuspid valve*.

**Figure 2:**
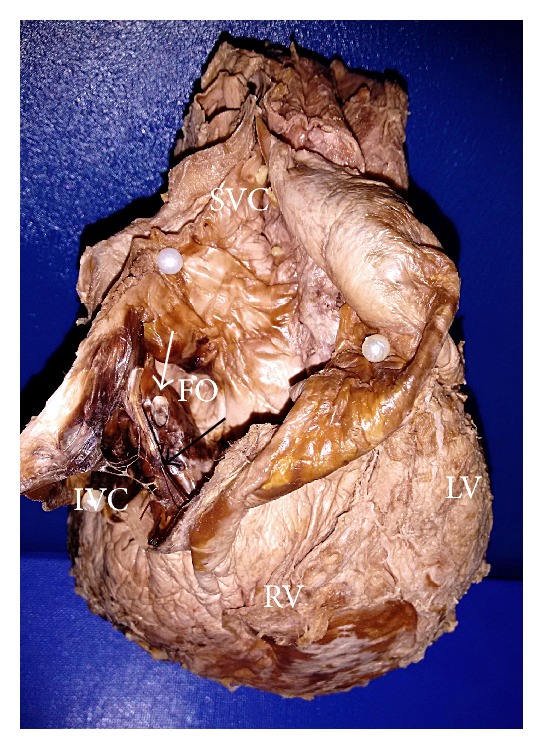
Dissection of right atrium showing Chiari network in the valve of inferior vena cava (*long black arrow*) and associated with the remnant of left venous valve observed as a membranous structure with single fenestration over the fossa ovalis (*short white arrow*).* SVC: superior vena cava*,* IVC: inferior vena cava*,* FO: fossa ovalis*,* RV: right ventricle*, and* LV: left ventricle*.

**Figure 3:**
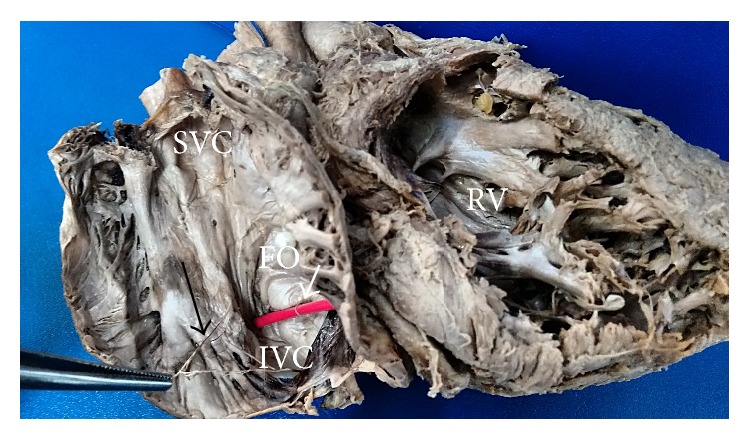
Right atrium dissection showing Chiari network as a reticular network of fine strands (*long black arrow*) attached to the valve of inferior vena cava and the right atrial wall. The remnant of left venous valve was observed as single strand over the fossa ovalis (*short white arrow*) which is highlighted by a red probe.* SVC: superior vena cava*,* IVC: inferior vena cava*,* FO: fossa ovalis*, and* RV: right ventricle*.

Out of those 3 specimens, Chiari network was observed as fenestrated membranous structure in 2 specimens (Figures 1 and 2) and reticular network of fine strands in 1 specimen (Figure 3) at the level of valve of inferior vena cava and extending to various sites of right atrium. The fenestrations varied from 10 (Figure 2) to 27 (Figure 1) in number. The network had the primary attachment over the valve of inferior vena cava in all the 3 specimens; however its further attachment extended to the orifice of coronary sinus in 1 specimen (Figure 1) and to the right atrial wall close to limbus fossa ovalis in other 2 specimens (Figures 2 and 3). The Chiari network was associated with left venous valve in 2 specimens, of which one is characterized as a single strand over the fossa ovalis (Figure 3) and the other is characterized as a membrane with single fenestration over the fossa ovalis (Figure 2). The findings are tabulated as below (Table 1).

### 3.2. Remnant of the Left Venous Valve

**Figure 4:**
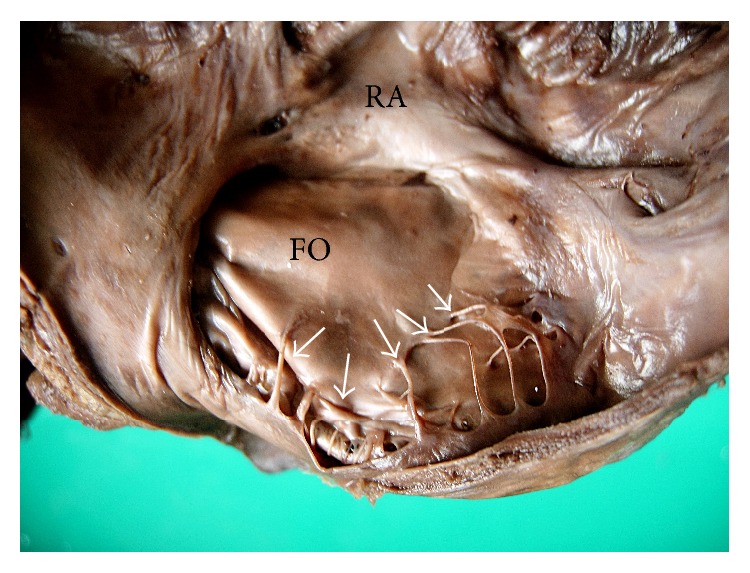
Interior of right atrium (RA) showing the remnant of left venous valve (*white arrows*) as multiple fine strand-like structures over the fossa ovalis (FO).

**Figure 5:**
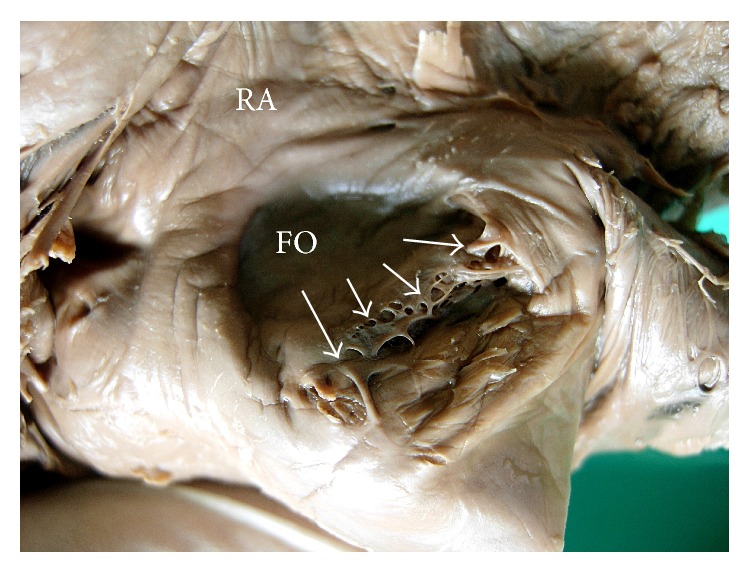
Interior of right atrium (RA) showing the remnant of left venous valve (*white arrows*) as trabecular membranous structure over the fossa ovalis (FO).

Out of 6 specimens with remnant of left venous valve, 2 were associated with Chiari network (Figures 2 and 3). Its characteristic morphology varied from fibrous strands like structures in 3 specimens (as in Figure 4) and trabecular membranous structure in 3 specimens (as in Figure 5).

## 4. Discussion

### 4.1. Embryological Basis

During development of heart, when the right horn of sinus venosus is incorporated into the primitive atrium to form the smooth part of right atrium, its entrance, the sinoatrial orifice is guarded by two muscular folds, the right and left valve of sinus venosus.

The cranial portion of the right venous valve is indicated as crista terminalis and its caudal portion forms the valve of inferior vena cava (Eustachian's valve) and valve of coronary sinus (Thebesian valves). The left venous valve blends with the right side of the interatrial septum [6].

During involution of these valves, the tissue undergoes fenestration so that a network may be formed from remnants that usually disappear. Incomplete resorption of right venous valve leads to Chiari network, which is described as a meshwork of thread-like strands connecting the edges of inferior vena cava and coronary sinus valves with crista terminalis [4] and with additional attachment to the wall of the right atrium or the interatrial septum [7].

If the left venous valve fuses with right aspect of interatrial septal complex incompletely; it remains free, leading to incomplete resorption by apoptosis. Thereby, the remnant of the left venous valve is found to be adherent to the superior portion of atrial septum or the fossa ovalis [8].

### 4.2. Incidence

Its incidence varies from 1.5 to 3% [3]. In the present study, the incidence of Chiari network is 3.75%. Most of the studies in the literature emphasize the incidence of Chiari network, diagnosed by echocardiographic findings. There are very few studies showing incidence of Chiari network in cadaveric hearts [2].

The incidence of left venous valve was observed in 6 out of 80 specimens (7.5%) in the present study. To the best of our knowledge, apart from case reports [5], there is no echocardiographic study that had been reported about the incidence of left venous valve remnant. Regarding the incidence in cadaveric heart, only one study reported that it was observed as a trabecular network in fossa ovalis in 3 out of 100 cadaveric hearts [2].

### 4.3. Characteristic Features

Chiari network is characterized as reticular network of fine strands attached to right atrium [3] or as membranous fenestration [9]. In the present study, it was observed as fenestrated membranous structure in 2 specimens and as reticular network of fine strands over the valve of inferior vena cava in 1 specimen.

This morphology is essential to identify the Chiari network in echocardiography. It is often observed as web-like structure with a variable number of thread-like components with characteristic whip-like motion within the right atrium moving with each contraction of the heart [10].

Regarding the morphology of left venous valve remnant, there is only one study that described it as a trabecular remnant over the fossa ovalis. In the present study, this remnant was noticed as fibrous strands in 3 specimens and as trabecular membranous structure in 3 specimens.

### 4.4. Clinical Significance of Chiari Network

Since Chiari network is considered as a remnant of right venous valve, it often prefers the pattern of fetal circulation, thus directing the blood flow towards the foramen ovale. This favors the persistence of patent foramen ovale thus creating cyanosis [1], atrial septal aneurysm, and paradoxical embolism from right to left atrium resulting to thromboembolic manifestations [3]. The network is associated with patent foramen ovale in 80% of cases [11]. However, in the present study Chiari network was not associated with patent foramen ovale.

Chiari network may create turbulent blood flow leading to thrombus formation. The fibers of the network are sometimes torn during life and may break free. The fenestrated types may rarely remove emboli from the circulation, but this is purely by chance, and further emboli are likely to reach the lung [2]. However, some authors [3, 12] believed that Chiari network acts as a congenital filter for inferior vena cava and may help with preventing massive pulmonary embolisms through filtration of blood.

Cardiac catheter can be entrapped by strands of Chiari network during an attempt to close the atrial septal defect [9, 13]. It can mimic right atrial thrombi, right heart vegetations, flail tricuspid leaflet [14], valve disruption, and ruptured chordae tendineae of tricuspid apparatus [15], a pedunculated right heart tumor [16] which may require surgical interventions. Hence the knowledge of Chiari network is essential to reach the correct diagnosis and can prevent unwanted surgery for a benign lesion.

In spite of its benign nature, Chiari network can be associated with infective endocarditis [17, 18], tricuspid atresia [19], fibroelastic papilloma [20], fetal hydrops [21], atrial septal aneurysm [1], atrial fibrillation [2], paradoxical embolism [11], Behcet's disease [22], and platypnea-orthodeoxia with atrial septal hypertrophy [23]. It may create an additional heart sound. Hence, the clinicians should be aware that Chiari network is not always a harmless structure [21]; the possible association of pathological conditions should be promptly scrutinized.

Chiari network can be more accurately diagnosed by transoesophageal echocardiography than the transthoracic echocardiography [7]. The requirement for surgical correction of Chiari network depends on the course and nature of the abnormality. In some cases, they require surgical correction and in some they have self-limiting progress [21] and can be treated medically with anticoagulants and close monitoring of the patient.

### 4.5. Clinical Significance of Left Venous Valve Remnant

The difficulty encountered during cardiac catheterization had been reported in 3 cases. In spite of its rarity, the knowledge of left venous valve remnant is mandatory for the successful device closure of atrial septal defect [5].

## 5. Conclusion

The incidence of Chiari network and left venous valve in the present study is 3.75% and 7.5%, respectively. Chiari network was observed as a fenestrated membranous structure in 2 specimens and a reticular network in 1 specimen, with variable extension to coronary sinus opening and right atrial wall. The remnant of left venous valve was observed as multiple fine strands in 3 specimens and trabecular structure in 3 specimens.

In spite of their rarity and being considered as benign, both Chiari network and remnant of left venous valve should be no longer considered as always-harmless structures. Therefore, the knowledge about their morphology and clinical manifestations is mandatory for the clinicians to reach for correct diagnosis, to achieve success in interventional procedures, and to anticipate the possible complications, so that surgical or medical management can be done at a right time.

## Figures and Tables

**Table 1:** Features of Chiari network and associated left venous valve remnant.

Specimen number	Characteristic feature	Number of fenestrations	Primary attachment	Extent to	Feature of the associated left venous valve remnant
1	Fenestrated membranous structure	27	Valve of inferior vena cava	Coronary sinus orifice	(Not associated)

2	Fenestrated membranous structure	10	Valve of inferior vena cava	Right atrial wall	Membranous structure with single fenestration

3	Reticular network of fine strands	—	Valve of inferior vena cava	Right atrial wall	Single strand over fossa ovalis
